# News and Events of the Week

**Published:** 1910-06-25

**Authors:** 


### News and Events of the Week.

The Senate of London University has appointed Lord Justice Moulton, F.R.S., a member of the council of the Lister Institute of Preventive Medicine.

The annual garden-party and distribution of prizes to students of the Medical School by Professor Howard Marsh at Guy's Hospital will take place on Thursday, July 7, at 3.15 p.m.

Mr. George Gwynn Bird, M.R.C.S., of Paddington, Fellow of the British Gynæcological Society, late chloroformist at the Female Lock Hospital, senior resident medical officer at St. Mary's Hospital, etc., who died on May 14, left estate valued at £9,349 gross and £9,230 net.

The annual prize-giving of the London (Royal Free Hospital) School of Medicine for Women took place on Thursday, June 23, at 3 p.m., when Dr. Miers, F.R.S., Principal of the University, distributed the medal, prizes, and certificates gained during the session. Mrs. Garrett Anderson, M.D., presided.

The New Palace Steamers P.S. *Koh-i-Noor* commenced her Saturday afternoon "Husband Boat" trips on Saturday, the 18th inst., and on Sunday, the 19th, she made her first trip this season to Deal. These trips mark the opening of the full service of sailings of the *Royal Sovereign* and *Koh-i-Noor*, particulars of which will be found in our advertisement columns. The special trains from St. Pancras call at a number of stations in the North of London, and are a great convenience to passengers residing in those districts, particulars of which can be obtained from Mr. T. E. Barlow, Director and Manager, 50 King William Street, London, E.C.

At the Quarterly Court of the Governors of the London Hospital, held on Wednesday, June 1, Mr. Hurry Fenwick was elected Professor of Urology on the recommendation of the Medical Council. We also learn that Mr. Hurry Fenwick was unanimously elected President of the ensuing Congress of Urology by the Council of the International Association of Urology at their last meeting in Paris. This International Association, of which Professor Guyon is the President, holds a Congress every third year. Its membership is limited and confined to experts in this particular branch of surgery. They are elected by ballot from among the prominent members of the different Urological Societies of Europe and America.

The third International Congress on School Hygiene will be held in Paris from August 2 to 7, 1910. The Minister of Public Instruction in France is the official head of this Congress. The Committee strongly urges all those associated with the second Congress, held in London in 1907, and others to attend the Paris Congress. On receipt of the subscription fee of £1 a card of membership will be forwarded, entitling the subscriber to all the privileges of the Congress and copies of the transactions. Subscriptions may be paid to the Secretaries of the Organising Committee of Great Britain and Ireland, 90 Buckingham Palace Road, S.W., and all information about the Paris Congress will be forwarded on application to them. The Committee is making arrangements for the English delegates to stay at superior-class hotels during the Congress, and for this purpose they have secured special terms for travelling and hotel accommodation (first class £6 2s., second £5 12s. inclusive). A very interesting and attractive programme has been arranged.

Marlborough's "Travellers' Esperanto Manual of Conversation" (6d. net) has just been published by the well-known firm of 51 Old Bailey, E.C. It is one of the "self-taught" series, and is practically a handbook of Esperanto which may be recommended to those who wish to study the new volapuk.

Mr. Michael Carteighe, of Goring-on-Thames, for fourteen years President of the Pharmaceutical Society, one of the founders of the Institute of Chemistry, has left estate valued at £61,753 gross and £48,282 net. He left £2,000 to the Pharmaceutical Society of Great Britain for the Society's general fund.

The opening ceremony of the extension of the Harrogate Baths will be performed on Saturday, July 2, 1910, by the Mayor (Councillor A. Boyd-Carpenter, B.A.). All members of the medical profession are cordially invited. The Corporation has issued an extremely attractive pamphlet guide, giving full particulars of the baths.

The Great Eastern Railway Company's new illustrated "Tourist Guide to the Continent," published at the price of 6d., has just been issued. Among its features are particulars of new tours, *via* Holland, in North Germany, including the Harz Mountains and Thuringian Mountains; in South Germany of the less-known side valleys of the Rhine; in Belgium, *via* Antwerp, of fresh tours in the Ardennes and old Flemish cities, and a series of Continental maps. A chapter on "Tourists' Travel Talk," a vocabulary in English, French, and German, has been added. The little book will prove a valuable guide to professional men who intend to take their holiday on the Continent.

We have received from Messrs. Eyre & Spottiswoode (Bible Warehouse), Ltd., a copy of the Memorial Prayer Book published in commemoration of his late Majesty King Edward VII. The book is elegantly bound in polished roan, with gilt side and back, and contains an illuminated title-page and four photogravures, viz. portrait of his late Majesty, the Lying-in-State at Buckingham Palace, the Lying-in-State at Westminster Hall, service at St. George's Chapel, Windsor, with three memorial services: (1) The special forms of service used in all churches on the day of the funeral; (2) form of service in Westminster Hall; (3) order of the service in St. George's Chapel, Windsor (by special permission). The price is 4s. 6d. net.

The election of four new members of the Council of the Royal College of Surgeons will take place on Thursday, July 7 next. There are four vacancies, and the ballot will be open from 3 to 5 o'clock p.m. for those Fellows who wish to record their votes by personal attendance at the college. The following are candidates: Charles Barrett Lockwood (Fellow 1881), St. Bartholomew's, retiring from the Council in rotation; Bilton Pollard (Fellow 1881), University, nominated by John Tweedy, L. A. Dunn, William Thorburn; Charles Alfred Ballance, M.V.O. (Fellow 1882), St. Thomas's, nominated by John Tweedy, Bernard Pitts, W. H. A. Jacobson; James Ernest Lane (Fellow 1882), St. Mary's, nominated by Herbert W. Page, A. T. Norton, V. Warren Low; John Bland-Sutton (Fellow 1884), Middlesex, nominated by Andrew Clark, A. Boyce Barrow, Bilton Pollard.

